# Supplementary material for: Betting on Non-Invasive Brain Stimulation to Treat Gambling Disorder: A Systematic Review and Meta-Analysis
Source: Brain Sci. 2023 Apr 21;13(4):698. doi: 10.3390/brainsci13040698 (PMC10136786; doi:10.3390/brainsci13040698)
Supplement: Supplementary file 1 [file brainsci-13-00698-s001.zip › brainsci-2321085-supplementary.pdf]

Supplementary Materials – Section A: Research strategy

Search Databases	Search algorithm	Records retrieved
**Pubmed**	("transcranial magnetic stimulation"[All Fields] OR "TMS"[All Fields] OR "repetitive transcranial magnetic stimulation"[All Fields] OR "rTMS"[All Fields] OR "theta burst stimulation"[All Fields] OR "TBS"[All Fields] OR "deep transcranial magnetic stimulation"[All Fields] OR "dTMS"[All Fields] OR "deepTMS"[All Fields] OR "transcranial direct current stimulation"[All Fields] OR "tDCS"[All Fields] OR "transcranial electrical stimulation"[All Fields] OR "tES"[All Fields] OR "transcranial alternating current stimulation"[All Fields] OR "tACS"[All Fields] OR "transcranial current stimulation"[All Fields] OR "tCS"[All Fields] OR "non invasive brain stimulation"[All Fields] OR "NIBS"[All Fields] OR "brain stimulation"[All Fields]) AND ("pathological gambling"[All Fields] OR "gamblers"[All Fields] OR "gambling"[All Fields] OR "gambling disorder"[All Fields] OR "GD"[All Fields]))	335
**EMBASE**	('transcranial magnetic stimulation'/exp OR 'transcranial magnetic stimulation' OR 'tms'/exp OR tms OR 'repetitive transcranial magnetic stimulation'/exp OR 'repetitive transcranial magnetic stimulation' OR rtms/exp OR rtms OR 'theta burst stimulation'/exp OR 'theta burst stimulation' OR tbs/exp OR tbs OR 'deep transcranial magnetic stimulation'/exp OR 'deep transcranial magnetic stimulation' OR dtms/exp OR dtms OR deeptms/exp OR deeptms OR 'transcranial direct current stimulation'/exp OR 'transcranial direct current stimulation' OR tdcs/exp OR tdcs OR 'transcranial electrical stimulation'/exp OR 'transcranial electrical stimulation' OR 'tes'/exp OR tes OR 'transcranial alternating current stimulation'/exp OR 'transcranial alternating current stimulation' OR tacs/exp OR tacs OR 'transcranial current stimulation'/exp OR 'transcranial current stimulation' OR tcs/exp OR tcs OR 'non invasive brain stimulation'/exp OR 'non invasive brain stimulation' OR nibs/exp OR nibs OR 'brain stimulation'/exp OR 'brain stimulation') AND ('pathological gambling'/exp OR 'pathological gambling' OR gamblers/exp OR gamblers OR gambling/exp OR gambling OR 'gambling disorder'/exp OR 'gambling disorder' OR 'gd'/exp OR gd)	688
**Web of Science**	(((TS=('transcranial magnetic stimulation') OR TS=(tms) OR TS=('repetitive transcranial magnetic stimulation') OR TS=(rtms) OR TS=('theta burst stimulation') OR TS=(tbs) OR TS=('deep transcranial magnetic stimulation') OR TS=(dtms) OR TS=(deaptms) OR TS=('transcranial direct current stimulation') OR TS=(tdcs) OR TS=(‘'transcranial electrical stimulation') OR TS=(tes) OR TS=('transcranial alternating current stimulation') OR TS=(tacs) OR TS=('transcranial current stimulation’) OR TS=(tcs) OR TS=('non invasive brain stimulation') OR TS=(nibs) OR TS=('brain stimulation')) AND (TS=('pathological gambling') OR TS=(gamblers) OR TS=(gambling) OR TS=('gambling disorder') OR TS=(gd))))	400
**Scopus**	((TITLE-ABS-KEY (“transcranial magnetic stimulation”) OR TITLE-ABS-KEY (tms) OR TITLE-ABS-KEY (“repetitive transcranial magnetic stimulation”) OR TITLE-ABS-KEY (rtms) OR TITLE-ABS-KEY (“theta burst stimulation”) OR TITLE-ABS-KEY (tbs) OR TITLE-ABS-KEY (“deep transcranial magnetic stimulation”) OR TITLE-ABS-KEY (dtms) OR TITLE-ABS-KEY (deeptms) OR TITLE-ABS-KEY (“transcranial direct current stimulation”) OR TITLE-ABS-KEY (tdcs) OR TITLE-ABS-KEY (“transcranial electrical stimulation”) OR TITLE-ABS-KEY (tes) OR TITLE-ABS-KEY (“‘transcranial alternating current stimulation”) OR TITLE-ABS-KEY (tacs) OR TITLE-ABS-KEY (“transcranial current stimulation”) OR TITLE-ABS-KEY (tcs) OR TITLE-ABS-KEY (“non invasive brain stimulation”) OR TITLE-ABS-KEY (nibs) OR TITLE-ABS-KEY (”brain stimulation”)) AND (TITLE-ABS-KEY (”'pathological gambling”) OR TITLE-ABS-KEY (gamblers) OR TITLE-ABS-KEY (gambling) OR TITLE-ABS-KEY (“gambling disorder”) OR TITLE-ABS-KEY (gd)))	3.614

Table S1. Details on the search strategy in the four screened databases.

Section B: Data extraction notes

Cardullo et al. 2019 [103] – as post-treatment, we included measures at Day 60. Data were taken from the abstract, standard errors were transformed into standard deviations.

Del Mauro et al. preprint [98] – MATE scores were used to measure craving and BDI depression. Data were taken from Table 2.

Gay et al. 2017 [102] – We included the VAS of craving before and after stimulation since it was the only measure in which we had scores before and after NiBS. Data were taken from Table 2 ("Cue-induced craving").

Pettorusso et al. 2020 [104] – T4 included post-treatment data (after intensive plus maintenance phases), data were taken from Table 2. We included PG-YBOCS and BDI as measures of craving and depressive symptoms, respectively.

Rosenberg et al. 2013 [105] – We included the VAS scores for craving and the HDRS for depressive symptoms. Mean, and SDs were calculated from Table 2.

Salerno et al. 2022 [106] – We included PG-YBOCS and HAMD scores and calculated mean and standard deviations from Table 1.

Zack et al. 2016 [81] – We asked for data by email, and the authors sent us data from the VAS administered before (Time 1) and after stimulation (Time 2).

Section C: Sensitivity analyses

Correlation	SMCC	CI 95%	p-value
Corr = .25	-0.609	-1.0875, -0.1297	.012
Corr = .75	-0.835	-1.434, -0.236	.006

Table S2. Sensitivity analysis results on craving scores effects.

Correlation = values established between pre- and post-measurement variances (0.25, 0.75) (analyses in the main text were run setting a 0.5 correlation); SMCC = standardized mean change (effect size); CI = confidence interval.

Section D: Risk of bias details for pre-post studies (Tables S3 – S7)

Table S3. Cardullo et al., 2019			
**The National Institutes of Health (NIH) quality assessment tool for before-after (Pre-Post) study with no control group**<br>**Website:** https://www.nhlbi.nih.gov/health-topics/study-quality-assessment-tools			
Major Components	Response options		
1. Was the study question or objective clearly stated?	Yes	No	Cannot Determine/ Not Applicable/ Not Reported
2. Were eligibility/selection criteria for the study population prespecified and clearly described?	Yes	No	Cannot Determine/ Not Applicable/ Not Reported
3. Were the participants in the study representative of those who would be eligible for the test/service/intervention in the general or clinical population of interest?	Yes	No	Cannot Determine/ Not Applicable/ Not Reported
4. Were all eligible participants that met the prespecified entry criteria enrolled?	Yes	No	Cannot Determine/ Not Applicable/ Not Reported
5. Was the sample size sufficiently large to provide confidence in the findings?	Yes	No	Cannot Determine/ Not Applicable/ Not Reported
6. Was the test/service/intervention clearly described and delivered consistently across the study population?	Yes	No	Cannot Determine/ Not Applicable/ Not Reported
7. Were the outcome measures prespecified, clearly defined, valid, reliable, and assessed consistently across all study participants?	Yes	No	Cannot Determine/ Not Applicable/ Not Reported
8. Were the people assessing the outcomes blinded to the participants' exposures/interventions?	Yes	No	Cannot Determine/ Not Applicable/ Not Reported
9. Was the loss to follow-up after baseline 20% or less? Were those lost to follow-up accounted for in the analysis?	Yes	No	Cannot Determine/ Not Applicable/ Not Reported
10. Did the statistical methods examine changes in outcome measures from before to after the intervention? Were statistical tests done that provided p values for the pre-to-post changes?	Yes	No	Cannot Determine/ Not Applicable/ Not Reported
11. Were outcome measures of interest taken multiple times before the intervention and multiple times after the intervention (i.e., did they use an interrupted time-series design)?	Yes	No	Cannot Determine/ Not Applicable/ Not Reported
12. If the intervention was conducted at a group level (e.g., a whole hospital, a community, etc.) did the statistical analysis take into account the use of individual-level data to determine effects at the group level?	Yes	No	Cannot Determine/ Not Applicable/ Not Reported
**Quality Rating**	Good	Fair	Poor
Additional Comments (If Poor, please state why):			

Table S4. Del Mauro et al. preprint			
**The National Institutes of Health (NIH) quality assessment tool for before-after (Pre-Post) study with no control group**<br>**Website:** https://www.nhlbi.nih.gov/health-topics/study-quality-assessment-tools			
Major Components	Response options		
1. Was the study question or objective clearly stated?	Yes	No	Cannot Determine/ Not Applicable/ Not Reported
2. Were eligibility/selection criteria for the study population prespecified and clearly described?	Yes	No	Cannot Determine/ Not Applicable/ Not Reported
3. Were the participants in the study representative of those who would be eligible for the test/service/intervention in the general or clinical population of interest?	Yes	No	Cannot Determine/ Not Applicable/ Not Reported
4. Were all eligible participants that met the prespecified entry criteria enrolled?	Yes	No	Cannot Determine/ Not Applicable/ Not Reported
5. Was the sample size sufficiently large to provide confidence in the findings?	Yes	No	Cannot Determine/ Not Applicable/ Not Reported
6. Was the test/service/intervention clearly described and delivered consistently across the study population?	Yes	No	Cannot Determine/ Not Applicable/ Not Reported
7. Were the outcome measures prespecified, clearly defined, valid, reliable, and assessed consistently across all study participants?	Yes	No	Cannot Determine/ Not Applicable/ Not Reported
8. Were the people assessing the outcomes blinded to the participants' exposures/interventions?	Yes	No	Cannot Determine/ Not Applicable/ Not Reported
9. Was the loss to follow-up after baseline 20% or less? Were those lost to follow-up accounted for in the analysis?	Yes	No	Cannot Determine/ Not Applicable/ Not Reported
10. Did the statistical methods examine changes in outcome measures from before to after the intervention? Were statistical tests done that provided p values for the pre-to-post changes?	Yes	No	Cannot Determine/ Not Applicable/ Not Reported
11. Were outcome measures of interest taken multiple times before the intervention and multiple times after the intervention (i.e., did they use an interrupted time-series design)?	Yes	No	Cannot Determine/ Not Applicable/ Not Reported
12. If the intervention was conducted at a group level (e.g., a whole hospital, a community, etc.) did the statistical analysis take into account the use of individual-level data to determine effects at the group level?	Yes	No	Cannot Determine/ Not Applicable/ Not Reported
**Quality Rating**	Good	Fair	Poor
Additional Comments (If Poor, please state why):			

Table S5. Pettorruso et al., 2020			
**The National Institutes of Health (NIH) quality assessment tool for before-after (Pre-Post) study with no control group**<br>**Website:** https://www.nhlbi.nih.gov/health-topics/study-quality-assessment-tools			
Major Components	Response options		
1. Was the study question or objective clearly stated?	Yes	No	Cannot Determine/ Not Applicable/ Not Reported
2. Were eligibility/selection criteria for the study population prespecified and clearly described?	Yes	No	Cannot Determine/ Not Applicable/ Not Reported
3. Were the participants in the study representative of those who would be eligible for the test/service/intervention in the general or clinical population of interest?	Yes	No	Cannot Determine/ Not Applicable/ Not Reported
4. Were all eligible participants that met the prespecified entry criteria enrolled?	Yes	No	Cannot Determine/ Not Applicable/ Not Reported
5. Was the sample size sufficiently large to provide confidence in the findings?	Yes	No	Cannot Determine/ Not Applicable/ Not Reported
6. Was the test/service/intervention clearly described and delivered consistently across the study population?	Yes	No	Cannot Determine/ Not Applicable/ Not Reported
7. Were the outcome measures prespecified, clearly defined, valid, reliable, and assessed consistently across all study participants?	Yes	No	Cannot Determine/ Not Applicable/ Not Reported
8. Were the people assessing the outcomes blinded to the participants' exposures/interventions?	Yes	No	Cannot Determine/ Not Applicable/ Not Reported
9. Was the loss to follow-up after baseline 20% or less? Were those lost to follow-up accounted for in the analysis?	Yes	No	Cannot Determine/ Not Applicable/ Not Reported
10. Did the statistical methods examine changes in outcome measures from before to after the intervention? Were statistical tests done that provided p values for the pre-to-post changes?	Yes	No	Cannot Determine/ Not Applicable/ Not Reported
11. Were outcome measures of interest taken multiple times before the intervention and multiple times after the intervention (i.e., did they use an interrupted time-series design)?	Yes	No	Cannot Determine/ Not Applicable/ Not Reported
12. If the intervention was conducted at a group level (e.g., a whole hospital, a community, etc.) did the statistical analysis take into account the use of individual-level data to determine effects at the group level?	Yes	No	Cannot Determine/ Not Applicable/ Not Reported
**Quality Rating**	Good	Fair	Poor
Additional Comments (If Poor, please state why):			

Table S6. Rosenberg et al., 2013			
**The National Institutes of Health (NIH) quality assessment tool for before-after (Pre-Post) study with no control group**<br>**Website:** https://www.nhlbi.nih.gov/health-topics/study-quality-assessment-tools			
Major Components	Response options		
1. Was the study question or objective clearly stated?	Yes	No	Cannot Determine/ Not Applicable/ Not Reported
2. Were eligibility/selection criteria for the study population prespecified and clearly described?	Yes	No	Cannot Determine/ Not Applicable/ Not Reported
3. Were the participants in the study representative of those who would be eligible for the test/service/intervention in the general or clinical population of interest?	Yes	No	Cannot Determine/ Not Applicable/ Not Reported
4. Were all eligible participants that met the prespecified entry criteria enrolled?	Yes	No	Cannot Determine/ Not Applicable/ Not Reported
5. Was the sample size sufficiently large to provide confidence in the findings?	Yes	No	Cannot Determine/ Not Applicable/ Not Reported
6. Was the test/service/intervention clearly described and delivered consistently across the study population?	Yes	No	Cannot Determine/ Not Applicable/ Not Reported
7. Were the outcome measures prespecified, clearly defined, valid, reliable, and assessed consistently across all study participants?	Yes	No	Cannot Determine/ Not Applicable/ Not Reported
8. Were the people assessing the outcomes blinded to the participants' exposures/interventions?	Yes	No	Cannot Determine/ Not Applicable/ Not Reported
9. Was the loss to follow-up after baseline 20% or less? Were those lost to follow-up accounted for in the analysis?	Yes	No	Cannot Determine/ Not Applicable/ Not Reported
10. Did the statistical methods examine changes in outcome measures from before to after the intervention? Were statistical tests done that provided p values for the pre-to-post changes?	Yes	No	Cannot Determine/ Not Applicable/ Not Reported
11. Were outcome measures of interest taken multiple times before the intervention and multiple times after the intervention (i.e., did they use an interrupted time-series design)?	Yes	No	Cannot Determine/ Not Applicable/ Not Reported
12. If the intervention was conducted at a group level (e.g., a whole hospital, a community, etc.) did the statistical analysis take into account the use of individual-level data to determine effects at the group level?	Yes	No	Cannot Determine/ Not Applicable/ Not Reported
**Quality Rating**	Good	Fair	Poor
Additional Comments (If Poor, please state why): On patient was treated twice, inclusion/eligibility criteria of included participants and follow-ups were not clear			

Table S7. Salerno et al. 2022			
**Q. The National Institutes of Health (NIH) quality assessment tool for before-after (Pre-Post) study with no control group**<br>**Website:** https://www.nhlbi.nih.gov/health-topics/study-quality-assessment-tools			
Major Components	Response options		
1. Was the study question or objective clearly stated?	Yes	No	Cannot Determine/ Not Applicable/ Not Reported
2. Were eligibility/selection criteria for the study population prespecified and clearly described?	Yes	No	Cannot Determine/ Not Applicable/ Not Reported
3. Were the participants in the study representative of those who would be eligible for the test/service/intervention in the general or clinical population of interest?	Yes	No	Cannot Determine/ Not Applicable/ Not Reported
4. Were all eligible participants that met the prespecified entry criteria enrolled?	Yes	No	Cannot Determine/ Not Applicable/ Not Reported
5. Was the sample size sufficiently large to provide confidence in the findings?	Yes	No	Cannot Determine/ Not Applicable/ Not Reported
6. Was the test/service/intervention clearly described and delivered consistently across the study population?	Yes	No	Cannot Determine/ Not Applicable/ Not Reported
7. Were the outcome measures prespecified, clearly defined, valid, reliable, and assessed consistently across all study participants?	Yes	No	Cannot Determine/ Not Applicable/ Not Reported
8. Were the people assessing the outcomes blinded to the participants' exposures/interventions?	Yes	No	Cannot Determine/ Not Applicable/ Not Reported
9. Was the loss to follow-up after baseline 20% or less? Were those lost to follow-up accounted for in the analysis?	Yes	No	Cannot Determine/ Not Applicable/ Not Reported
10. Did the statistical methods examine changes in outcome measures from before to after the intervention? Were statistical tests done that provided p values for the pre-to-post changes?	Yes	No	Cannot Determine/ Not Applicable/ Not Reported
11. Were outcome measures of interest taken multiple times before the intervention and multiple times after the intervention (i.e., did they use an interrupted time-series design)?	Yes	No	Cannot Determine/ Not Applicable/ Not Reported
12. If the intervention was conducted at a group level (e.g., a whole hospital, a community, etc.) did the statistical analysis take into account the use of individual-level data to determine effects at the group level?	Yes	No	Cannot Determine/ Not Applicable/ Not Reported
**Quality Rating**	Good	Fair	Poor
Additional Comments (If Poor, please state why):			

Section E: Participants' inclusion and exclusion criteria across papers

References	Inclusion criteria	Exclusion criteria
Cardullo et al. [103]	CUD in comorbidity with GD	History of other psychiatric diseases, alcohol, and other SUDs (excluding cocaine and tobacco), personality disorders, and unstable medical illness
Del Mauro et al. [98]	Diagnosis of GD	No psychiatric comorbidity
Gay et al. [102]	Diagnosis of GD	Comorbidity with other SUDs (excluding nicotine), psychiatric disorders, unstable psychiatric medications
Martinotti et al. [100]	Age 18-65 years; current diagnosis of SUDs or GD	No comorbid diagnosis of bipolar disorder, schizophrenia, or other psychotic disorders; no history of seizures or neurological disorders; no current use of pro convulsant drugs; stable pharmacotherapy
Pettorruso et al. [104]	Medically healthy, drug-free, or under a stable pharmacological regimen	Severe psychiatric comorbidity, current alcohol, or SUDs comorbidity (except nicotine), any neurological disorder
Rosenberg et al. [105]	Diagnosis of GD	SUDs
Salerno et al. [106]	GD for at least one year and a PG-YBOCS score ≥16	No comorbidity for mood disorders, the presence of a risk of seizure or epilepsy, implanted devices, metal in the brain, and neurological disorders
Sauvaget et al. [101]	Right-handed, GD diagnosis	Comorbidity with SUDs, AUD, cognitive impairment, neurological disease, epilepsy brain injury, brain surgery, medications likely to modify the seizure threshold
Soyata et al. [99]	GD diagnosis, age between 18 and 65, right-handed, drug-free	Current diagnosis of major depressive disorder, current or previous AUD or SUDs (including tobacco), psychotic disorders, neurological disorder, use of psychotropic medications
Zack et al. [81]	Diagnosis of GD	Axis I comorbidity, current use of drugs or psychoactive medications

Table S8. Inclusion and exclusion criteria of participants within the analyzed papers.

Alcohol Use Disorder (AUD); Cocaine Use Disorder (CUD); Diagnostic and Statistical Manual (DSM); Gambling Disorder (GD); Male (M); Pathological Gambling Yale-Brown Obsessive Compulsive Scale (PG-YBOCS); Substance Use Disorders (SUDs).
